# Long-Term Survival and Outcome in Children Admitted to Kilifi District Hospital with Convulsive Status Epilepticus

**DOI:** 10.1155/2014/643747

**Published:** 2014-01-30

**Authors:** Agnes Prins, Eddie Chengo, Victor Mung'ala Odera, Manish Sadarangani, Claire Seaton, Penny Holding, Greg Fegan, Charles R. Newton

**Affiliations:** ^1^Centre for Geographic Medicine Research-Coast (CGMRC), Kenya Medical Research Institute (KEMRI), P.O. Box 230, Kilifi 80108, Kenya; ^2^Department of Paediatrics, University of Oxford, Level 2, Children's Hospital, Oxford OX3 9DU, UK; ^3^Department of Paediatrics, University of British Columbia & BC Children's Hospital, 4480 Oak Street, Vancouver, BC, Canada V6H 3V4; ^4^Department of Paediatrics, John Radcliffe Hospital, Oxford University Hospitals NHS Trust, Headington, Oxford OX3 9DU, UK; ^5^International Centre for Behavioral Studies, P.O. Box 34307, Mombasa 80118, Kenya; ^6^Centre for Clinical Vaccinology and Tropical Medicine, Churchill Hospital, Old Road, Oxford OX3 7LJ, UK; ^7^Neurosciences Unit, Institute of Child Health, University College London, 30 Guilford Street, London WC1N 1EH, UK; ^8^Department of Psychiatry, University of Oxford, Oxford OX3 7JX, UK

## Abstract

*Objectives*. The incidence of convulsive status epilepticus (CSE) is high in Africa but the long-term outcome is unknown. We examined the neurocognitive outcome and survival of children treated for CSE in a Kenyan hospital 3 to 4 years after discharge. *Methods*. The frequency and nature of neurological deficits among this group of children were determined and compared to a control group. The children were screened with the Ten Questions Questionnaire for neurodevelopmental impairment if alive and those that screened positive were invited for further assessment to determine the pattern and extent of their impairment. A verbal autopsy was performed to determine the cause of death in those that died. *Results*. In the 119 cases followed-up, 9 (8%) died after discharge, with the majority having seizures during their fatal illness. The 110 survivors (median age 5 years) had significantly more neurological impairments on the screening compared to 282 controls (34/110 (30.9%) versus 11/282 (3.9%), OR = 11.0, 95% CI 5.3–22.8). Fifteen percent of the cases had active epilepsy. *Conclusions*. This study demonstrates the considerable burden of CSE in African children. Strategies to manage children with CSE that are acceptable to the community need to be explored to improve the longer-term outcome.

## 1. Introduction

Convulsive status epilepticus (CSE) is the most common neurological emergency in children. It is associated with an increase in mortality and neurological sequelae, but the frequency of these outcomes varies across the world [1]. The incidence and severity of CSE are presumed to be greater in developing countries; however, data are sparse [2]. The incidence of CSE in the West is estimated at 17–23/100,000 children per year [3, 4], highest in children below the age of one year [5]. Overall, between 10% and 20% of children with epilepsy will have at least one episode of CSE during the course of the disease, most occurring in the first few years of epilepsy onset [6], but in sub-Saharan Africa CSE occurs in 25% of people with active convulsive epilepsy [7]. In the United Kingdom the short-term (within 30 days) mortality after CSE is 3–5% and 5–8% in children admitted to intensive care. The long-term mortality after a first episode of CSE is variable, ranging from 5% to 17% [8]. In Western survivors of CSE, less than 15% have focal neurological deficits, cognitive impairment, and behavioral problems; however, 13 to 74% of CSE patients develop epilepsy [8].

There are few published data on CSE in children living in Africa, despite the fact that the prevalence of epilepsy is higher than in Europe and North America [9]. In Kenya the prevalence of active epilepsy is 11/1,000 and the incidence is 187/100,000/year in children aged 6–9 years [10]. In the first reported study from Africa, the incidence of CSE in children was at least twice that of Western countries [10], with a case fatality of 15% in hospital, and 12% were discharged with neurological deficits, mostly motor impairment.

In this study we report on the long-term outcome of these children, 3 to 4 years after discharge. The frequency, nature, and extent of neurological deficits were determined and compared to that of a control group. We also assessed the long-term mortality and investigated causes of death.

## 2. Materials and Methods

### 2.1. Study Sites

The study was conducted in an area within Kilifi District on the Kenyan coast that was mapped in 2000-2001 and has a Health and Demographic Surveillance System (HDSS) with regular (usually 4 monthly) community censuses. Kilifi District Hospital (KDH) is the only hospital in the area. Details of the study area have been described previously [11].

### 2.2. Participants

The study cohort was identified from a group of 388 children admitted to KDH with CSE in 2002 and 2003 [11]. Ninety-five percent were 2–9 years at the time of admission. Inclusion criteria for cases were (i) confirmed CSE (according to the International League Against Epilepsy criteria at the time of this study [12]), defined as any seizure lasting for 30 minutes or longer, or intermittent seizures lasting for greater than 30 minutes during which the patient did not regain consciousness, that is, Blantyre Coma Score (BCS) < 3 [13]; (ii) probable CSE, defined by the presence of any of the following criteria: convulsing on arrival at hospital; parenteral phenobarbital or phenytoin administered to stop convulsions after the failure of two doses of the first-line medication (diazepam, paraldehyde, or both); BCS < 3 on arrival at hospital and more than one convulsion in previous 30 minutes; BCS < 3 on arrival and >10 convulsions in previous 24 hours [11]. Availability of benzodiazepines and other antiepileptic drugs in the community is very limited, so it would not be expected that the BCS would be modified due to drug administration before arrival to hospital. A list of control children was randomly selected from the HDSS database, using the most recent census round conducted prior to recruitment. They were matched for age, sex, and area of residence.

### 2.3. Study Procedures

#### 2.3.1. Determination of Mortality and Screening for Impairment

Children were visited at home by a fieldworker fluent in the local language, Kigiriama. If the child had died, a verbal autopsy was conducted by a fieldworker trained in this technique to determine the cause of death [14]. In particular, evidence of epilepsy and convulsions during the agonal stages was determined. For the children that were alive the “Ten Questions Questionnaire” (TQQ) was administered to the parents or guardians to screen for neurological disability or impairment [15]. The TQQ has been validated in Kilifi for the detection of neurocognitive impairment and has a sensitivity of 71% and specificity of 98% for detecting moderate-or-severe motor impairment [15]. Children who screened positive on the TQQ were invited for further assessment at KDH to determine the pattern and extent of their sensorimotor or neurological impairments.

#### 2.3.2. Hospital Based Assessments

During further evaluation, a full birth and medical history was obtained, in addition to sociodemographic details. A thorough neurological examination was performed by a clinician trained in neurological examinations that included assessment of the cranial nerves and motor function to detect features such as spasticity, ataxia, and fine motor dysfunction [16]. Vision was assessed using a Sonksen-Silver chart for children aged 3(1/2) years and older [17], which provided a measure of distant and near acuity using Snellen 6 meter standard specifications. Hearing was assessed with a Kamplex screening audiometer (P.C. Werth, London, UK) in children older than 5 years. Air conduction at 500 Hz, 1000 Hz, 2000 Hz, and 4000 Hz in each ear was carried out according to the recommendations of British Society of Audiology [18]. Hearing impairment was classified according to the lowest audible tone and defined as mild impairment 40–50 dB, moderate impairment 51–60 dB, severe impairment 61–70 dB, and profound impairment > 70 dB. A 20–30-minute electroencephalogram (EEG) with photic stimulation and hyperventilation was performed in those with reported seizures, to identify epileptic discharges and to help classify the seizures [19].

### 2.4. Data Analysis

Data were collected using standard forms and entered into an electronic database using FoxPro (version 9, Microsoft, Redmond, WA, USA) and statistical analysis was performed using SPSS (version 17.0, SPSS, Chicago, IL, USA). The cases and controls were compared with respect to neurological impairments on either the TQQ or hospital based assessments, using Pearson Chi-square analysis and Fisher's exact test if an expected cell value was <5. Differences between the groups were estimated by a calculation of the odds ratio (OR) and these are reported with 95% confidence intervals (95% CI).

### 2.5. Ethical Considerations

Ethical approval was obtained from the KEMRI National Ethical and Scientific committees in Nairobi (SSC-1074). Informed consent was obtained from the parents or caretakers of the participants.

## 3. Results 

### 3.1. Description of Study Participants

**Figure 1:**
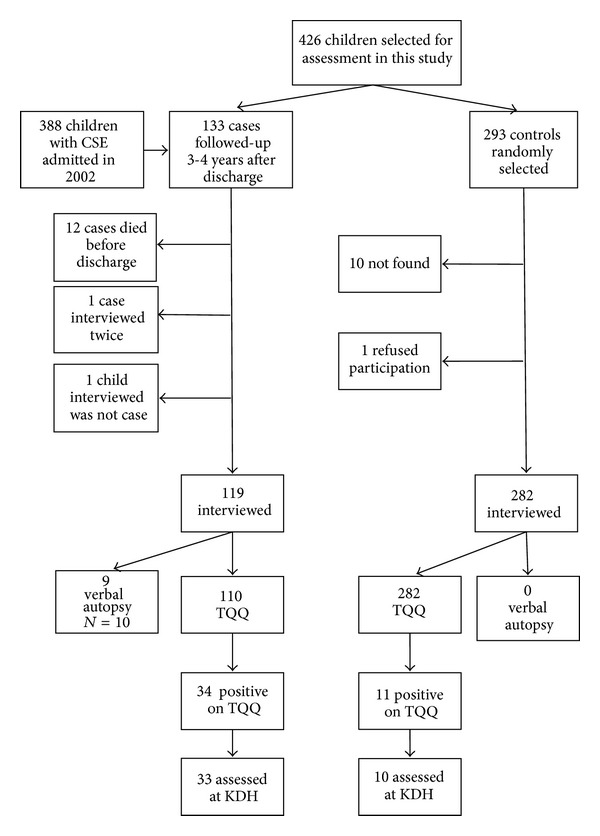


In the period 2002-2003 a total of 388 children were admitted to hospital who fulfilled the criteria of confirmed (*n* = 155) or probable (*n* = 233) CSE [11]. One hundred and nineteen children were followed up during a 5-month period in 2006 and their caretakers were identified and interviewed (Figure 1). The follow-up duration was 3-4 years. The TQQ was administered to the caretakers of 110 cases and the verbal autopsy to the caretakers of 9 cases who had died. There were no significant differences in the clinical characteristics between the 119 cases recruited and the 269 not recruited regarding age, sex, seizure type at admission, and impairment at discharge. Many children had multiple discharge diagnoses. Significantly more children that were not recruited had a discharge diagnosis of non-TB meningitis compared to the children who were recruited (Table 1). A total of 293 possible controls were randomly selected for interview, at least two controls for each case, of which we were able to interview 282 caretakers (Figure 1).

### 3.2. Mortality

On follow-up, 9 (7.6%) cases had died after discharge. Eight had a fever during the illness that led to death, and 8 experienced generalized seizures during their fatal illness. Seven children had experienced both fever and generalized seizures. None of the deceased children were taking AEDs at the time of their death.

### 3.3. Screening with the Ten Questions Questionnaire

The caretakers of 110 cases and 282 controls were interviewed with the TQQ (Figure 1). The median age was 5 years (range: 3 to 14 years), and 176 (44.9%) were male. Significantly more cases screened positive on the TQQ than controls (34/110 (30.9%) versus 11/282 (3.9%), OR = 11.0, 95% CI 5.3–22.8). In particular, significantly more cases screened positive to questions about vision, hearing, movement, cognition, and speech (Table 2). The most common neurological problem reported by the parents of cases was the child “appearing mentally backward, dull, or slow.” Of the 110 cases, 12 (10.9%) had neurological deficits at time of discharge from hospital after the CSE episode. Of the 34 cases with one or more impairments reported on the TQQ, 10 had had an impairment observed at discharge, with 9 of them a motor impairment.

### 3.4. Assessments

#### 3.4.1. Neurological Examination

Of the 43 children who were assessed at the clinic, there were no significant differences in the demographic characteristics or birth history of the controls compared to the cases. On neurological examination 15 cases (45.5%) and 1 control (10.0%) had neurological deficits. Using the denominators of the children screened with the TQQ, cases were significantly more likely to have deficits compared to controls (15/110 (13.6%) versus 1/282 (0.4%), *P* < 0.001). This difference was mostly due to higher prevalence of motor, speech, and visual impairment and epilepsy (Table 3).

#### 3.4.2. Vision and Hearing

In the visual screening 20 cases and 4 controls could not be given a score, due to inability of the child to match the letters on the practice board (19/24), inability to follow the instructions of the fieldworker (4/24), or inappropriate age for the test (1/24). These included the 3 children who were thought to have a visual problem during the neurological examination. None of the children that were assessed were found to have a visual impairment. Similarly on the hearing screening 19 cases and 4 controls were unable to do the test; most of them could not follow the instructions given to them (20/23), and 8 were too young to complete the test. Two cases and 1 control were found to have a hearing impairment, but these numbers are too small to draw firm conclusions with regard to the difference between the two groups.

#### 3.4.3. Epilepsy

Seven of the 110 children (6.4%) alive in this cohort had a discharge diagnosis of epilepsy after their admission for CSE. Twelve cases (10.9%) were using antiepileptic drugs: either phenobarbital, carbamazepine, or phenytoin. Of the 43 children assessed at KDH, 9 did not cooperate sufficiently to undergo an EEG and data for 9 children were not collected due to technical problems. Thus EEG was performed on 19 cases and 6 controls. Epileptiform activity was observed in 7 cases (21.2%) and 1 control (10.0%) which was mostly focal temporal or focal extratemporal features. After reviewing the history 16 cases and 1 control were diagnosed with epilepsy, defined as recurrent unprovoked seizures [12].

## 4. Discussion

This study demonstrates that children who are discharged from hospital following CSE in a rural area of Kenya have a poorer developmental outcome compared to community controls. In total 30% of the cases had an impairment reported on the TQQ, with cognitive, motor, and speech domains being the most affected. On neurological examination significantly more cases had motor, speech, and vision impairment compared to the controls. Overall 15% of the cases had active epilepsy. Furthermore there was considerable mortality in the children discharged following CSE, with a majority (8/9) having seizures during their terminal illness.

The prevalence of neurodevelopmental impairments reported in this study is higher than that reported in the West, where it is less than 15% (excluding epilepsy) [8]. One possible explanation is that these children often present with a longer duration of seizures, since they have limited access to medical facilities in this region [2]. The longer a seizure persists, the more likely is the seizure to be unresponsive to antiepileptic drugs, the higher the mortality, and the worse the outcome in survivors [20]. In most Western studies the underlying cause of the CSE appears to be the main determinant of neurological impairment [1, 21]. However, in this study the number of children having a face-to-face assessment was low, so we were unable to examine this.

Long-term follow-up of this cohort demonstrates that the prevalence of epilepsy following an episode of CSE increases over time. At discharge 6% of children were diagnosed with epilepsy, while after three years the prevalence had doubled. This highlights that prolonged acute symptomatic seizures may contribute to the high incidence of epilepsy in this region [2].

### 4.1. TQQ and Assessments

Some of the impairments reported on the TQQ, that is, motor and speech impairment, were confirmed during the hospital based neurological assessments, but not all of them. The evaluation of sensory deficits (vision/hearing) was hampered by difficulties in engaging the children in the procedures. Although visual impairment was not found in the visual screening, most of the cases were not able to match the letters on the practice board and might not have understood the instructions due to cognitive problems. In testing the hearing, a lot of children were unable to point or use the tools or could not sit still. In other assessments a significant proportion was found to be uncooperative as well. While these findings support the high frequency of cognitive problems reported by parents, without specific measures of behaviour or cognition we are not able to validate either the sensory measures used or determine the true cause of the children's lack of cooperation.

### 4.2. Longer-Term Mortality

The mortality of children who survived to discharge from hospital was 8% after 3-4 years. If the children that died before discharge are added, it amounts to 23% of the children that were admitted to hospital with CSE had died within 4 years of their admission. This is higher than in the West, where the cumulative long-term mortality after a first episode of CSE ranges from 5% to 17% [8]. In most of the cases, the child appeared to die with a seizure and a febrile illness suggesting that children who have suffered status epilepticus previously are more susceptible to febrile status epilepticus.

### 4.3. Treatment Gap

None of the children who had died were using AEDs. The inadequate treatment of epilepsy probably plays an important role in the outcome of CSE. Nonadherence for AEDs is a risk factor for CSE in children with epilepsy. It is estimated that in the Kilifi district 70% of epilepsy patients are not receiving appropriate medication [22]. Besides the high costs of AEDs, the parent's perception of epilepsy might also explain this treatment gap, since seizures are thought to be caused by spiritual causes and thus not amenable to treatment by AEDs [23]. Traditional healers have a profound influence on the treatment of epilepsy, who are not likely to refer a child with seizures to hospital when their treatment fails. Furthermore, in this area distance to a health facility has been an important factor in treatment seeking [24]. To reduce the seizures and the associated impairments in children, intervention should focus on educating the parents in recognizing CSE as a treatable illness and forge communication between health care workers and traditional healers to improve treatment programmes for these patients.

### 4.4. Limitations to the Study

We were not able to compare the mortality of cases and controls since the controls were recruited from the most recent census round data, which did not include the children that had died. We could not ensure that the controls had not had any seizures, and caretakers of 5 controls reported their child had fits sometimes. We were not able to administer neuropsychological tests to estimate the degree of cognitive impairments in the children. Although the TQQ detects developmental delay, this needs to be verified with comprehensive assessments. The high number of “probable” CSE in the baseline study makes it difficult to compare the outcome to Western studies, where different criteria for CSE are used. These definitions were used, since in this context the history of seizure duration is very unreliable.

## 5. Conclusions

In this rural African population, a single episode of CSE requiring hospital admission is associated with significant developmental sequelae and high mortality. Nearly a quarter of children in our hospital based sample died following CSE, either as inpatients or after discharge from hospital. An additional one-third was identified as having developmental deficits after three to four years, with epilepsy developing in a considerable proportion. This data is likely to underestimate the total burden of CSE, since not all children with the condition will present to hospital. The results of this study emphasise the need for education of parents and carers, as well as community healthcare workers, to enable early, aggressive treatment of seizures that should improve longer-term outcome. In addition policy makers and health care planners should be aware of the need to ensure an adequate supply of AEDs as well as increased access to facilities where children can receive adequate supportive care.

## Figures and Tables

**Table 1:** Clinical characteristics of children recruited for follow-up compared to children not recruited.

Demographic and clinical characteristics at the time of admission for CSE	Found (*N* = 119)	Not found (*N* = 269)	*P* value
Male	52 (43.9%)	137 (50.9%)	0.189
Age in years, median (range)	2.2 (0.3–10.3)	2.1 (0.1–12.4)	—
Generalised convulsion (either initial or secondary)^§^	94 (79.7%)	189 (71.1%)	0.077
Focal onset seizure	44 (37.0%)	107 (39.8%)	0.602
Neurological impairment at discharge^§^ Discharge diagnosis^¥^	15 (12.7%)	31 (14.7%)	0.619
Sepsis*	2 (1.7%)	10 (3.7%)	0.358
Malaria	79 (66.4%)	173 (64.3%)	0.693
Febrile convulsion	41 (34.5%)	68 (25.3%)	0.064
Non-TB meningitis	4 (3.4%)	36 (13.4%)	0.003
Encephalopathy of unknown cause	14 (11.8%)	39 (14.5%)	0.470
Anaemia	19 (16.0%)	56 (20.8%)	0.264

^¥^Some children had multiple diagnoses at discharge.

*Differences between “found” and “not found” with Fisher's exact test.

^§^Number may not be exact due to missing values.

**Table 2:** Responses to Ten Questions Questionnaire in cases and controls.

	Cases *N* = 110	Controls *N* = 282	*P* value
	Yes	Yes	
(1) Child had delay in sitting, standing, or walking	10 (9.1%)	3 (1.1%)	<0.001
(2) Child has difficulty seeing	4 (3.6%)	0 (0%)	0.006
(3) Child appears to have difficulty hearing	11 (10.0% )	7 (2.5% )	0.001
(4) Child understands what you are saying	101 (91.8%)	280 (99.6%)	<0.001
Child does not understand what you are saying	9 (8.2%)	1 (0.4%)	<0.001
(5) Child has difficulty walking or moving his/her arms or has weakness and/or stiffness in arms or legs	13 (11.8%)	1 (0.4%)	<0.001
(6a) Child sometimes has fits, becomes rigid, or loses consciousness^¥^	—	5 (1.8%)	Not done
(6b) Since admission to KDH, child has had fits, has become rigid, or has lost consciousness^§^	14 (12.7%)	—	Not done
(7) Child learns to do things like other children his/her age	102 (93.6%)	282 (100%)	<0.001
Child does not learn to do things like other children his/her age	7 (6.4%)	0 (0%)	<0.001
(8) Child speaks	99 (90.0%)	281 (99.6%)	<0.001
Child does not speak	11 (10.0%)	1 (0.4%)	<0.001
(9) Child's speech is different from normal	13 (11.8%)	1 (0.4%)	<0.001
(10) Child appears mentally backward, dull, or slow	14 (12.7%)	1 (0.4%)	<0.001
Other serious health problems	17 (15.7%)	28 (10.0%)	0.111
Child is attending school regularly, no. (%)	48 (43.6%)	143 (50.9%)	0.197

^*¥*^Question only for controls.

^§^Question only for cases.

**Table 3:** Neurological deficits on assessment.

Neurological deficits on assessment	Cases *N* = 33	Controls *N* = 10	*P* value*
Total screened with TQQ	*N* = 110	*N* = 282	
Any neurological deficit, no. (% of total screened with TQQ)	15 (13.6%)	1 (0.4%)	<0.001
Speech impairment	11 (10.0%)	0	<0.001
Motor impairment	11 (10.0%)	0	<0.001
Vision impairment	3 (2.7%)	0	0.022
Hearing impairment	1 (0.9%)	0	0.281
Cognitive impairment	1 (0.9%)	1 (0.4%)	0.483
Epilepsy	16 (14.5%)	1 (0.4%)	<0.001

*Differences between cases and controls with Fisher's exact test.
